# Secondhand Smoke Exposure, Indoor Smoking Bans and Smoking-Related Knowledge in China

**DOI:** 10.3390/ijerph111212835

**Published:** 2014-12-11

**Authors:** Yue Jin, Ling Wang, Bo Lu, Amy K. Ferketich

**Affiliations:** 1 Division of Epidemiology, College of Public Health, The Ohio State University, 1841 Neil Ave., Columbus, OH 43210, USA; E-Mails: jin.177@osu.edu (Y.J.); wang.3347@osu.edu (L.W.); 2 Division of Biostatistics, College of Public Health, The Ohio State University, 1841 Neil Ave., Columbus, OH 43210, USA; E-Mail: lu.232@osu.edu

**Keywords:** clean-indoor air laws, public smoking bans, smoke-free environments, secondhand smoke, tobacco control policies/interventions

## Abstract

Although previous studies have provided strong evidence that Chinese individuals are exposed to secondhand smoke (SHS) and lack knowledge of its harmful effects, there has not been an in-depth exploration of the variability in exposure and knowledge by geographic region, occupation, and socioeconomic status. The objectives of this study were to examine: (1) the demographic factors associated with the level of knowledge of the harmful effects of smoking; (2) the factors related to implementation of in-home and workplace smoking bans; and (3) geographic differences in being exposed to SHS in government buildings, healthcare facilities, restaurants, public transportations, and schools. We used data from the 2010 Global Adult Tobacco Survey-China. Chi-square tests were used for statistical analysis. The results suggested that among Chinese citizens age 15 years and older, there is poor knowledge of the harmful effects of tobacco, and knowledge varies with region and socioeconomic status. Over three-quarters of the households had no smoking restrictions, and a large percentage of workers reported working in places with no smoking ban. In public places, exposure to SHS was high, particularly in rural areas and in the Southwest. These results suggest Chinese individuals are not well informed of smoking and SHS associated risks and are regularly exposed to SHS at home, work and public places.

## 1. Introduction

Secondhand smoke (SHS) exposure is known to cause adverse health outcomes among non-smokers [1]. Since there is no minimum threshold at which exposure acts to produce health effects, banning smoking in indoor places is a fundamental approach to protecting non-smokers against SHS exposure [1]. Previous studies have found that smoke-free laws in indoor places are effective at reducing SHS exposure [2,3]. In addition, knowledge about the harmful effects of SHS exposure is an important factor related in reducing SHS exposure. Several studies have indicated that people with better knowledge are more likely to protect themselves and others against SHS exposure by opening windows, establishing a smoking ban in the home, or keeping their children out of a smoking environment [4,5,6].

According to the World Health Organization (WHO), among the one billion smokers worldwide, 80% live in low- and middle-income countries [7]. In China, there are over 300 million smokers, which makes it the country with the world’s largest population of smokers [8]. Previous research has shown that tobacco use could cause premature death in more than one-third of males in China by 2030 [9].

Based on increasing awareness about the harmful effects of SHS exposure, many developed countries have made efforts to reduce SHS exposure in public places and in the home [10,11,12,13,14,15]. However, China’s efforts to protect people from SHS exposure in public places have not been as effective [8].

Although the Chinese Government ratified the WHO’s Framework Convention on Tobacco Control (FCTC) in 2005, 72% of non-smokers aged 15 and older were still regularly exposed to SHS in 2010, with more than half exposed on a daily basis [16]. Smoking restrictions in public places are usually imposed by public policy. China’s indoor smoking policies differ by region and are mostly only implemented in major cities [17]. Moreover, the policies do not include workplaces [17].

During the past decade, many studies have reported on SHS exposure in China [16,18,19,20,21,22,23]. In 2004, a cross-sectional survey, performed in six counties in China, found that only 6.3% of households had implemented a complete in-home smoking ban [21]. In 2010, a local household survey conducted in Guangdong Province, China suggested that more homes have complete smoking bans [22]. According to the 2010 Global Adult Tobacco Survey-China (GATS China), about 67.3% of adults reported someone smoked at home during past month [23]. King and colleagues used GATS data from 14 countries and examined SHS exposure by age and gender. They found that Chinese residents had relatively high exposure to SHS in the home and workplaces compared to other countries [19]. In other studies, awareness of the harmful effects of smoking and SHS exposure has been linked to a reduced risk of SHS exposure [4,5,6]. While previous studies have provided strong evidence that Chinese individuals are exposed to SHS and lack knowledge of its harmful effects, there has not been an in-depth exploration of the variability in exposure and knowledge by geographic region, occupation, and socioeconomic status. Furthering this understanding may guide the future development of effective tobacco control programs.

We used data from 2010 Global Adults Tobacco Survey-China (GATS China) to examine: (1) the demographic factors associated with the level of knowledge of the harmful effects of smoking; (2) the factors related to implementation of in-home and workplace smoking bans; and (3) geographic differences in being exposed to SHS in government buildings, healthcare facilities, restaurants, public transportations, and schools.

## 2. Methods 

### 2.1. Study Population

We used data from the 2010 GATS in China, which is a nationally representative household survey of the population aged 15 years and older. As a component of Global Tobacco Surveillance System (GTSS), during 2008 and 2010 the GATS used a standardized method to monitor tobacco use and tobacco control conditions across 16 low-and-middle income countries, including China. The 2010 GATS China used a multi-stage stratified cluster sampling design to produce nationally representative data. In total, 13,354 respondents completed the survey interviews, which are in-person interviews administered by interviewers using hand-held devices. The overall response rate was 96%. Key findings from and additional details of the survey methodology of GATS China are available in the GATS China fact sheet [23].

### 2.2. Measures 

Knowledge of the harmful effects of smoking was assessed with three core questions: “Based on what you know or believe, does smoking tobacco cause the following: Stroke (blood clots in the brain that may cause paralysis)/Heart attack/Lung cancer”? In addition, knowledge of the harmful effect of SHS was measured by questions: “Based on what you know or believe, does breathing smoke from other people’s cigarette cause the following: Heart disease in adult/Lung illness in children/lung cancer in adult”? Participants who answered all three questions correctly were defined as have good knowledge; those who answered any of two questions correctly were defined as having some knowledge, and the rest were defined as have little knowledge.

In-home smoking bans were assessed by the sampled participant’s reporting of smoking rules inside the home. Participants who reported that smoking was not allowed inside the home were classified as having a total smoking ban; those who reported smoking was not allowed but with exceptions to that rule were classified as having partial smoking ban; and those who reported that smoking was allowed or had no rules were defined as having no ban. Participants who reported working indoors were asked to describe the smoking policy at work. Workplace smoking rules were classified as a full ban, a partial ban, and no ban.

SHS exposure in public places was assessed by a set of questions that asked if participants had seen anyone smoking in government buildings, healthcare facilities, on public transportation, in restaurants and at schools during the past 30 days. Healthcare facilities were assessed by the questions that did anyone smoke inside of any “private/village health care facilities”, “hospital or community health care facilities” or “other health care facilities”. People who reported viewing smoking at any location above were defined as being exposed to SHS in public places.

Smoking status was defined by the question: “Do you currently smoke any tobacco product on a daily basis, less than daily, or not at all?” An adult who currently smoked any tobacco product daily or less than daily was defined as current smoker. Demographics variables included age (15–34 years; 35–44 years; 45–59 years; ≥60 years), gender, highest attained education level (“primary school” or less; “secondary school” or less; “high school/technical secondary school”; “college” or higher), region (North; Northeast; East; Mid-south; Southwest; Northwest), urban/rural area, career type (agriculture workers; equipment operator or technician; business or service industry employee; leader of organizations; medical and health personnel; teacher; other), and wealth index. Wealth index was measured by principal component analysis based on a set of questions about whether a household has certain items, including electricity, flush toilet, fixed telephone, cell phone, television, radio, refrigerator, car, moped/scooter/motorcycle, washing machine, and air conditioner/heater [20]. Based on the results of a principal component analysis, the score of first principal component was divided into quintiles to classify a person’s socioeconomic status [20].

### 2.3. Statistical Analysis

All statistical analyses were conducted using the survey functions in Stata 12.0 (Stata Corporation, College Station, TX, USA). The data were properly weighted using the personal weights, and the survey design features such as strata and clustering were accounted for in the analyses.

Descriptive statistics were calculated for the sample, by smoking status and gender. In addition, the knowledge level of the harmful effects of smoking and SHS exposure were assessed for the sample by demographic characteristics. The prevalence of in-home and workplace smoking bans were reported by household and geographic characteristics. In addition, the prevalence of viewing smoking in public places was estimated by geographic characteristics. Chi-square tests were used to analyze the differences in smoking and SHS related knowledge, smoking bans, and prevalence of viewing smoking in public places by corresponding dependent variables. Individuals who had incomplete information on wealth index variables, knowledge on smoking or SHS exposure, reported in-home or workplace smoking ban, or SHS exposure in public places were excluded from the corresponding analysis.

## 3. Results

Our study identified the factors related to better knowledge of the harmful effects of smoking and SHS exposure, smoking bans at home and workplaces and SHS exposure in public places. In addition to the existing studies on the topic, our study focused on geographic factors and socioeconomic status including education level, wealth index and career type.

Table 1 summarizes characteristics of 13,354 individuals who were representative of the 1,068,752,451 people aged 15 years and older in China. The weighted data suggests that 28.3% of individuals were current smokers, and approximately 95.8% of current smokers were male. More than half of males reported to be current smokers (52.9%); among females, the smoking prevalence was 2.4%. There was a difference in the age distribution between male and female smokers, with females being older than males. In addition, over half of the female smokers had an educational level lower or equal primary school. In contrast, the majority of the male smokers had an educational level greater than primary school. Moreover, the majority of the female smokers were agriculture workers and none of the female health personnel or teachers were smokers. Interestingly, more than one-third of female smokers were from Northeast China; however, less than 10% of male smokers were from that region.

**Table 1:** Characteristics of study population by smoking status and gender.

Characteristics		Current Smoker ^a^ (28.3%)											Non-smoker ^a^ (71.7%)							
		Male (95.8%)			Female(4.2%)		Total		*p* Value				Male(33.4%)	Female(66.6%)			Total		*p* Value	
**Age**									<0.001 *										<0.001 *	
15–34 Years		30.3			7.7		29.4						47.7	36.0			39.9			
35–44 Years		28.0			25.0		27.8						17.7	25.0			22.6			
45–59 Years		29.0			32.8		29.2						17.6	23.3			21.4			
≥60 Years		12.7			34.5		13.6						17.0	15.7			16.1			
**Education**									<0.001 *										<0.001 *	
≤Primary School		22.0			59.4		23.5						18.6	34.0			28.9			
≤Secondary School ^b^		44.8			24.0		44.0						39.1	36.2			37.2			
High School/Technical Secondary School ^b^		23.2			10.7		22.7						25.6	18.3			20.8			
≥College		10.0			5.9		9.8						16.7	11.4			13.2			
**Urban/Rural**									0.196										0.226	
Urban		42.4			50.8		42.8						49.3	46.4			47.4			
Rural		57.6			49.2		57.2						50.7	53.6			52.6			
**Region**									<0.001 *										0.018 *	
North		13.0			16.2		13.2						12.2	12.9			12.7			
Northeast		9.6			36.0		10.7						11.5	8.5			9.5			
East		28.4			18.2		27.9						34.3	30.5			31.8			
Mid-south		18.2			8.5		17.8						19.0	19.5			19.3			
Southwest		23.9			18.7		23.7						16.1	21.6			19.7			
Northwest		6.9			2.4		6.7						6.9	7.0			7.0			
**Wealth Index**									0.743										0.024 *	
Lowest		16.0			19.9		16.1						16.0	15.3			15.5			
Low		19.5			21.8		19.6						14.8	18.8			17.5			
Middle		20.7			18.5		20.6						19.9	20.6			20.4			
High		21.9			19.2		21.8						21.3	21.1			21.2			
Highest		21.9			20.6		21.9						28.0	24.2			25.4			
**Career**									<0.001 *										<0.001 *	
Agriculture workers		34.2			41.0		34.5						25.2	33.1			30.5			
Equipment operator/Technician		26.2			1.7		25.1						17.2	7.5			10.8			
Business/Service industry employee		16.8			10.5		16.5						13.0	16.5			15.3			
Leader of organizations		6.7			6.5		6.7						6.4	3.7			4.6			
Medical/Health personnel		1.0			0.0		1.0						1.7	2.1			1.9			
Teacher		0.8			0.0		0.8						1.7	2.0			1.9			
Other		14.3			40.4		15.4						34.8	35.1			35.0			

Notes: **^a^**: All percentages were weighted using personal weights, survey strata and primary sampling units (PSUs) information; **^b^**: Secondary school in China is equivalent to middle school or junior high school in the U.S. (usually cover grade 7–9); High School/Technical Secondary School is equivalent to senior high school in the U.S. (usually cover grade 10–12); *: *p* Value < 0.05.

The information in Table 2 relates knowledge to age, gender, educational level, career type and smoking status. Over half of the population had little knowledge of the harmful effects of smoking (56.8%) and SHS exposure (51.5%). In contrast, only about 22.1% of the target population had good knowledge of the harmful effects of smoking, and 24.6% had good knowledge related to SHS exposure. Non-smokers appeared to be more aware of the harm of smoking (*p* = 0.002) and SHS (*p* < 0.001) compare to smokers. Approximately 23.0% of non-smokers had good knowledge of smoking, and 25.6% had good knowledge of SHS. Among current smokers, 19.6% and 22.1% had good knowledge of smoking and SHS, respectively.

Younger individuals appeared to have better knowledge of the harmful effects of smoking (*p* = 0.004) and SHS exposure (*p* < 0.001). Near 60% of people aged between 15 and 34 had some or good knowledge of SHS exposure, while among people greater than 60 years old, only 32.5% had some or good knowledge of SHS exposure. In general, the awareness of the harmful effects of smoking (*p* = 0.045) and SHS exposure (*p* = 0.044) among females was not as good as it was among males. People who were better educated tended to be more aware of the harmful effects of smoking (*p* < 0.001) and SHS (*p* < 0.001). Among people had attended primary school or less, 70.8% and 75.1% had little knowledge about smoking and SHS exposure, respectively. In contrast, among people had attended college or higher, 64.6% and 77.1% had some or good knowledge of smoking and SHS, respectively. Moreover, nearly 70% of agriculture workers had little knowledge of the harmful effects of smoking and SHS. In contrast, medical/health personnel and teachers had better knowledge, with the majority of medical/health personnel having good knowledge.

**Table 2:** Knowledge of harm effects of smoking and SHS exposure.

Characteristics	Smoking Knowledge^ a^				SHS Exposure Knowledge^ a^			
	Little (56.8%)	Some (21.1%)	Good (22.1%)	*p* Value	Little (51.5%)	Some (23.9%)	Good (24.6%)	*p* Value
**Age**				0.004 *				<0.001 *
15–34 Years	54.1	24.0	22.0		40.9	32.1	27.0	
35–44 Years	58.3	21.1	20.6		50.8	23.3	26.0	
45–59 Years	57.3	19.0	23.7		58.3	18.6	23.1	
≥60 Years	61.1	16.8	22.1		67.5	13.3	19.2	
**Gender**				0.045 *				0.044 *
Male	55.3	21.6	23.1		50.2	24.0	25.8	
Female	58.7	20.3	21.0		52.8	23.8	23.4	
**Education**				<0.001 *				<0.001 *
≤Primary School	70.8	14.8	14.3		75.1	12.6	12.4	
≤Secondary School ^b^	59.0	20.9	20.1		52.4	24.4	23.2	
High School/Technical Secondary School ^b^	47.7	25.6	26.8		35.9	31.5	32.6	
≥College	35.4	27.2	37.4		22.9	34.3	42.8	
**Career**				<0.001 *				<0.001 *
Agriculture workers	68.8	16.2	15.0		69.5	15.6	14.9	
Equipment operator/Technician	52.4	24.3	23.3		44.5	30.8	24.7	
Business/Service Industry employee	58.6	21.9	19.5		47.1	28.6	24.3	
Leader of organizations	39.9	22.3	37.8		35.1	26.1	38.8	
Medical/Health personnel	20.2	23.9	55.8		17.2	20.5	62.3	
Teacher	39.2	26.5	34.3		21.9	31.4	46.7	
Other	51.7	23.3	25.0					
**Smoking Status**				0.002 *				<0.001 *
Non-smoker	55.2	21.7	23.0		49.6	24.8	25.6	
Current Smoker	61.3	19.1	19.6		56.3	21.6	22.1	

Notes: **^a^**: All analyses were weighted using personal weights, survey strata and PSUs information; **^b^**: Secondary school in China is equivalent to middle school or junior high school in the U.S. (usually cover grade 7–9); High School/Technical Secondary School is equivalent to senior high school in the U.S. (usually cover grade 10–12); *: *p* Value < 0.05.

Table 3 contains information on the implementation of indoor smoking bans in homes and at workplaces in China. In total, about 9.3% of the households had a full smoking ban, 12.1% had partial ban, and 78.6% of the households had no restrictions on smoking in home. Households in urban areas (*p* < 0.001) and higher income households (*p* < 0.001) had a higher prevalence of in-home smoking bans. The implementation of in-home smoking bans was not statistically different by regions (*p* = 0.142). At workplaces, the status of smoking bans did not differ by region or urban *vs.* rural areas. However, people who are equipment operators or technicians and people who work in business or the service industry were less likely to be protected by smoking bans at work. In contrast, medical/health personnel and teachers were more likely to report a full ban at work.

Among the various public places, the prevalence of viewing smoking in restaurants was the highest (89.4%), followed by government buildings (59.6%), healthcare facilities (38.8%), schools (37.7%) and public transportation vehicles (34.4%) (Table 4). The prevalence of exposure to smoking in healthcare facilities and on public transportation was higher in rural areas than in urban areas. About 67% of respondents reported seeing smoking in healthcare facilities in rural *vs.* 33% in urban areas; 42.3% of respondents were exposed to smoking in public transportation vehicles in rural areas *vs.* 27% in urban areas. Exposure to smoking at restaurants and schools was not different in rural and urban areas. In addition, the Southwest region had the worst smoking environment at government buildings, healthcare facilities and public transportation, followed by the Mid-south and the Northeast.

**Table 3:** Prevalence of smoking bans in home and at workplaces.

Characteristics	In-Home Smoking Ban (n = 13,308)^ a^					Workplace Smoking Ban (n = 4431)^ a^			
	Full (9.3%)	Partial (12.1%)	No Ban (78.6%)	*p* Value		Full (28.8%)	Partial (30.4%)	No Ban (40.8%)	*p* Value
**Urban/Rural**				<0.001 *					0.079
Urban	13.4	18.5	68.1			30.7	31.7	37.6	
Rural	5.8	6.7	87.6			25.5	28.3	46.2	
**Region**				0.142					0.064
North	10.7	17.6	71.7			39.4	26.6	34.0	
Northeast	9.6	15.5	74.8			28.9	33.6	37.5	
East	10.8	12.2	77.0			29.6	29.4	41.0	
Mid-south	5.1	7.9	87.0			28.2	28.1	43.7	
Southwest	6.8	10.0	83.3			18.0	37.4	44.6	
Northwest	17.9	14.6	67.5			29.5	27.4	43.1	
**Wealth Index**				<0.001 *					-
Lowest	7.7	4.2	88.1			-	-	-	
Low	5.9	6.1	88.0			-	-	-	
Middle	8.0	12.0	80.0			-	-	-	
High	10.3	15.3	74.4			-	-	-	
Highest	12.8	18.9	68.3			-	-	-	
**Career ^b^**									<0.001 *
Equipment operator/ Technician	-	-	-			26.9	30.8	42.3	
Business/ Service industry employee	-	-	-			23.6	26.3	50.1	
Leader of organizations	-	-	-			24.1	35.2	40.7	
Medical/ Health personnel	-	-	-			48.1	36.6	15.3	
Teacher	-	-	-			41.8	36.5	21.7	
Other	-	-	-			34.6	30.6	34.8	

Notes: **^a^**: All analyses were weighted using personal weights, survey strata and PSUs information; **^b^**: Agriculture workers were excluded from the analysis because they usually work outdoors; *: *p* Value < 0.05.

**Table 4:** Prevalence of viewing smoking in various public places.

Characteristics	Government Buildings (n = 1289)	Healthcare Facilities (n = 4713)	Public Transportation (n = 6569)	Restaurants (n = 5808)	Schools (n = 2449)
**Prevalence (%)**	**59.6**	**38.8**	**34.4**	**89.4**	**37.7**
**Urban/Rural**					
Urban	57.2	33.0	27.0	89.4	37.0
Rural	62.5	67.0	42.3	89.4	38.5
*p Value*	*0.524*	0.030 *	0.015 *	*0.993*	*0.686*
**Region**					
North	45.3	26.1	19.1	87.9	30.2
Northeast	66.3	38.3	31.6	88.9	28.4
East	55.2	32.3	29.9	91.6	39.1
Mid-south	53.4	41.2	37.6	87.4	39.1
Southwest	75.7	54.5	48.6	90.6	42.0
Northwest	46.2	32.9	28.4	85.1	38.5
*p Value*	0.017 *	0.011 *	0.024 *	*0.412*	*0.822*

Notes: All analyses were weighted using personal weights, survey strata and PSUs information; * *p* Value < 0.05.

## 4. Discussion

Our results indicate that, among Chinese citizens age 15 years and older, there is poor knowledge of the harmful effects of tobacco, and knowledge varies with region and socioeconomic status. As a consequence, perhaps, over three-quarters of households reported having no smoking ban and a substantial number of workers, especially equipment operators and business industry employees, reported that their workplaces had no restriction on smoking. In addition, SHS exposure in public places is a significant problem in China. Restaurants were the major places where people reported being exposed, followed by government buildings, health facilities, schools, and public transportation.

Article 8 from the WHO FCTC requires parties to implement and enforce smoke-free policies to protect people from exposure to tobacco smoke [24]. However, results from our study suggest that implementation and enforcement of smoke-free policies in China is in great need of improvement. In 2010, about 22.1% of people had good knowledge of smoking and health consequences, which was not that different from the level in 2002, three years before the FCTC was ratified in China [25]. The percentage of people with little knowledge of smoking effects in other low-and-middle income countries ranges from 5.1% in Egypt to 33.1% in India [20]. Given our finding that 56.8% of Chinese people have little knowledge of the harmful effects of smoking, we can conclude that Chinese adults are much less aware than individuals living in low- and middle-income countries. Although medical/health personnel and teachers appeared to have better knowledge than the general Chinese population, still, about 20% of health personnel and 40% of teachers had limited awareness of the harmful effects of smoking. Health personnel and teachers are responsible for educating and disseminating health-related knowledge to patients, students and others. Enhancing their understanding should be a priority because it could ultimately improve knowledge among others in China.

Knowing about the harmful effects of smoking and SHS exposure has been associated with adoption of in-home smoking bans; thus, this lack of knowledge that we found could partly explain our other finding that few homes had a complete in-home smoking ban [26]. The study found that 9.3% of households had full bans in home. Although this rate has been improved from the 6.3% in a 2004 survey, it is much lower than the 66% reported among Chinese Americans in New York City [21,27]. A previous study has shown that Chinese parents lack knowledge of the health risk of SHS exposure, and the main reasons for not banning smoking in home were the social acceptability of smoking and the predominant influence of male family members [28]. In developed countries, by changing the social norms and attitudes toward smoking, the prevalence of in-home SHS exposure has decreased [29]. Implementing smoking bans in public places can be one approach to change the social norms surrounding tobacco use and subsequently lead to more smoking bans at home. The smoke-free air legislation in New York City is an example. After implementation of the law, the adoption rate of in-home smoking ban increased significantly [27]. In addition, having smoking bans at workplaces has a positive influence on adopting smoking bans at home. The percentage of adults who report working in a smoking-free environment in other low-and-middle income countries, ranges from 35% in Bangladesh to 83% in Uruguay [30]. Our finding that few people worked in smoke-free environments may also explain the low percentage of smoke-free homes. Although the Chinese government has ratified the FCTC in 2005, no national smoking bans in public places have been implemented. At the city level, Beijing, Shanghai, Guangzhou, Hong Kong, and some smaller cities have passed indoor smoking bans in public places. However, the policies are ambiguous, exclude workplaces and are not supported with adequate public education and enforcement [17].

The finding of widespread secondhand exposure in public places also suggests the need for implementation and enforcement of clean indoor air laws. Nearly 90% of participants reported seeing smoking in restaurants, and smoking was reported to be seen in healthcare facilities, on public transportation and in schools. In general, the prevalence of SHS exposure in public places appeared to be higher in the Southwest, Northeast and rural areas. The high prevalence of SHS exposure in rural areas and Southwest China may be due to the imbalanced implementation of indoor smoking bans, cultural influences on smoking behavior and toleration of SHS exposure. China has a unique smoking culture. For instance, smoking and serving cigarettes is considered as good business manners; tobacco products are popular gifts of respect on both formal and informal occasions. Our finding that a majority of the business and service industry employees worked at places without any smoking restriction may also explain such smoking culture. In general, there is little social stigma against smoking, especially in rural areas, where people tend to have a lower educational level and a higher smoking prevalence [23]. Culturally tailored strategies are needed to address this problem by geographic region and within specific populations, such as people of lower socioeconomic status, lower educational level and people living in rural areas. However, China’s tobacco industry generates nearly 10% of government revenue each year, which makes the Chinese government itself the biggest stakeholder of such tobacco control strategies [31]. The Gross Domestic Product-first guideline of China, along with the widespread social acceptability of tobacco use and high smoking prevalence, may hinder the adoption and implementation of the tobacco control strategies [32].

Our study supports previous findings and provides updated information. We used nationally representative GATS data from China. The overall response rate was 96%, which indicates a good representation of the Chinese population. However, we relied on self-reported data on people’s smoking behavior, their in-home and workplace smoking bans, and exposure to secondhand smoke in public places. Although there is no information on biomarkers of cigarette smoking or exposure to secondhand smoke, these self-reported measures have been found to be valid and reliable in population-based studies [33]. In addition, there are only three questions to assess knowledge of the harmful effects of smoking or SHS exposure. It may limit our capability of assessing knowledge in a comprehensive way. Finally, because of the within-household sampling method used to select respondents, we were unable to associate personal characteristics, such as smoking behavior and knowledge of the harmful effects of smoking and SHS, to household smoking bans. Thus, in the future, it will be important to examine how knowledge of an individual relates to the choice to implement a household smoking ban.

## 5. Conclusions

In conclusion, this study suggests that Chinese individuals are not well informed of the risks associated with smoking and SHS and that they are regularly exposed to SHS at home, at work, and in other public places. Low knowledge and exposure are even more prevalent among people with a low socioeconomic status and those living in rural areas. The Chinese government needs to develop targeted educational and tobacco control programs to fit the cultural and social needs for Chinese citizens.
